# A role for macrophages under cytokine control in mediating resistance to ADI-PEG20 (pegargiminase) in ASS1-deficient mesothelioma

**DOI:** 10.1007/s43440-023-00480-6

**Published:** 2023-04-03

**Authors:** Melissa M. Phillips, Iuliia Pavlyk, Michael Allen, Essam Ghazaly, Rosalind Cutts, Josephine Carpentier, Joe Scott Berry, Callum Nattress, Shenghui Feng, Gunnel Hallden, Claude Chelala, John Bomalaski, Jeremy Steele, Michael Sheaff, Frances Balkwill, Peter W. Szlosarek

**Affiliations:** 1 Center for Cancer Biomarkers and Biotherapeutics, Barts Cancer Institute (BCI)-a Cancer Research UK Center of Excellence, Queen Mary University of London, John Vane Science Center, London, EC1M 6BQ UK; 2 Department of Medical Oncology, Barts Health NHS Trust, St Bartholomew’s Hospital, West Smithfield, London, EC1A 7BE UK; 3 Center for Tumor Microenvironment, Barts Cancer Institute (BCI)-a Cancer Research UK Center of Excellence, Queen Mary University of London, John Vane Science Center, London, EC1M 6BQ UK; 4 Centre for Haemato-Oncology, Barts Cancer Institute (BCI)-a Cancer Research UK Center of Excellence, Queen Mary University of London, John Vane Science Center, London, EC1M 6BQ UK; 5 Medicines and Healthcare Products Regulatory Agency (MHRA), London, UK; 6 Breast Cancer Now Toby Robins Research Centre, The Institute of Cancer Research, London, UK; 7 Polaris Pharmaceuticals, Inc., San Diego, CA 92121 USA; 8 Department of Histopathology, Barts Health NHS Trust, Royal London Hospital, London, E1 1BB UK

**Keywords:** Mesothelioma, ASS1, Macrophages, Arginine deprivation, ADI-PEG20

## Abstract

**Background:**

Pegylated arginine deiminase (ADI-PEG20; pegargiminase) depletes arginine and improves survival outcomes for patients with argininosuccinate synthetase 1 (ASS1)-deficient malignant pleural mesothelioma (MPM). Optimisation of ADI-PEG20-based therapy will require a deeper understanding of resistance mechanisms, including those mediated by the tumor microenvironment. Here, we sought to reverse translate increased tumoral macrophage infiltration in patients with ASS1-deficient MPM relapsing on pegargiminase therapy.

**Methods:**

Macrophage-MPM tumor cell line (2591, MSTO, JU77) co-cultures treated with ADI-PEG20 were analyzed by flow cytometry. Microarray experiments of gene expression profiling were performed in ADI-PEG20-treated MPM tumor cells, and macrophage-relevant genetic “hits” were validated by qPCR, ELISA, and LC/MS. Cytokine and argininosuccinate analyses were performed using plasma from pegargiminase-treated patients with MPM.

**Results:**

We identified that ASS1-expressing macrophages promoted viability of ADI-PEG20-treated ASS1-negative MPM cell lines. Microarray gene expression data revealed a dominant CXCR2-dependent chemotactic signature and co-expression of VEGF-A and IL-1α in ADI-PEG20-treated MPM cell lines. We confirmed that ASS1 in macrophages was IL-1α-inducible and that the argininosuccinate concentration doubled in the cell supernatant sufficient to restore MPM cell viability under co-culture conditions with ADI-PEG20. For further validation, we detected elevated plasma VEGF-A and CXCR2-dependent cytokines, and increased argininosuccinate in patients with MPM progressing on ADI-PEG20. Finally, liposomal clodronate depleted ADI-PEG20-driven macrophage infiltration and suppressed growth significantly in the MSTO xenograft murine model.

**Conclusions:**

Collectively, our data indicate that ADI-PEG20-inducible cytokines orchestrate argininosuccinate fuelling of ASS1-deficient mesothelioma by macrophages. This novel stromal-mediated resistance pathway may be leveraged to optimize arginine deprivation therapy for mesothelioma and related arginine-dependent cancers.

**Supplementary Information:**

The online version contains supplementary material available at 10.1007/s43440-023-00480-6.

## Introduction

Malignant pleural mesothelioma (MPM) remains an incurable asbestos-linked cancer with a 5 year survival of around 10% that has persisted despite two decades of clinical investigation [1]. Ipilimumab and nivolumab was approved over 2 years ago as the first-line combination immunotherapy extending the median overall survival of patients with mesothelioma to 18 months compared with 14 months for the standard of care chemotherapy, namely platinum and pemetrexed [2–4]. Moreover, while the incidence of mesothelioma is plateauing in many Western countries, cases are expected to increase over the next several decades in developing nations which continue to use asbestos [5].

To improve therapeutic options for patients, we have focused specifically on arginine deprivation as a novel antimetabolite strategy for mesothelioma and related arginine-auxotrophic cancers [6, 7]. Arginine, a multifunctional amino acid involved in protein and small molecule synthesis such as nitric oxide and polyamines, is non-essential for normal cells but essential for cancer cells harboring urea cycle (UC) enzyme dysfunction [8–10]. In particular, loss of the urea cycle enzyme argininosuccinate synthetase 1 (ASS1) redirects the arginine precursor aspartate for enhanced nucleotide synthesis leading to accelerated tumorigenesis and increased sensitivity to arginine deprivation and anti-folate chemotherapy [11, 12]. Furthermore, the therapeutic enzyme, pegylated arginine deiminase (ADI-PEG20; pegargiminase), selectively degrades arginine into citrulline and ammonia and inhibits a wide range of ASS1-negative tumor models in vivo [13–15].

In the clinic, pegargiminase monotherapy (ADAM trial) provided a modest progression-free survival benefit versus best supportive care in patients with newly diagnosed or relapsed ASS1-deficient MPM (3.2 months versus 2.0 months; *p* = 0.03) [16]. Furthermore, pegargiminase combined with platinum and pemetrexed (ADIPemCis; TRAP Trial) chemotherapy documented a 100% disease control rate and an encouraging overall survival of 14 months in patients with ASS1-deficient MPM [17]. Notably, screening for ASS1 loss in the TRAP trial enriched for patients with the highly aggressive non-epithelioid, biphasic and sarcomatoid, subtypes characterized by historical median survivals of 4–8 months.

Resistance to arginine-catabolizing enzymes, such as pegargiminase, is a significant hurdle that needs to be addressed to optimize arginine deprivation for anticancer therapy. Notwithstanding the role of drug immunogenicity due to anti-drug antibodies in the case of ADI-PEG20 which is derived from *Mycoplasma hominus*, non-immunogenic resistance mechanisms are also in operation [18]. These include the re-expression of ASS1 via promoter demethylation or c-Myc displacement of HIF-1α, and autophagy or the ‘self-eating’ of cellular organelles to provide arginine, thereby bypassing depletion of the amino acid [19–21]. Notably, in the TRAP dose-expansion cohort of patients with MPM treated with up to six cycles of ADIPemCis chemotherapy, subsequent tumor progression on maintenance ADI-PEG20 was linked to CD68^pos^ ASS1^pos^ macrophage infiltration using paired biopsies (*p* = 0.026) [22]. Indeed, this macrophage influx—rather than re-expression of tumoral ASS1—was the main observation in patients with thoracic cancers progressing on pegargiminase (*p* = 0.007).

Based on the tumor progression biopsies, we hypothesized that macrophages—which constitute up to 30% of the total cell population of mesothelioma—may be recruited by MPM cells as a novel mechanism of resistance to pegargiminase [23, 24]. To delineate the relationship between tumor associated macrophages (TAMs) and MPM cells under arginine restriction, we employed co-culture assays, gene expression profiling arrays, and gene pathway analysis. Further validation was sought using plasma analyses from patients with MPM on pegargiminase therapy and murine tumor models.

## Materials and methods

### Cell lines

The cell lines were obtained from American Type Culture Collection (MSTO, H226, and H28), Prof. Pasi Janne (2591), and Prof. Ken O’Byrne (JU77). MSTO, 2591 and JU77 cell lines are ASS1 negative; H28 and H226 are ASS1 positive; and a JU77 cell line overexpressing ASS1 was also used as a control [6, 11]. All cell lines were Short Tandem Repeat (STR) profiled to ensure quality and integrity. ASS1 tumoral overexpression was performed as described previously [11]. Cells were maintained in endotoxin-free RPMI 1640 medium (Gibco, 61870010) supplemented with 10% heat-inactivated fetal bovine serum (FBS) (Gibco, 16140071) at 37 °C in a humidified atmosphere of 5% CO_2_.

### Generation of macrophages from human peripheral blood mononuclear cells (PBMC)

‘Buffy cones’ of lymphocyte-rich peripheral blood from healthy human donors were purchased from the National Blood Service and stored at 4 ℃ to maintain cell viability. Blood was diluted in PBS followed by adding Ficoll-Paque^TM^PLUS (GE Healthcare, 17144002). The tubes were then spun at 1200 rpm for 45 min at 4 ℃ and set to decelerate slowly without a break. The lymphocyte-rich white layer was then aspirated from the middle and mixed with sterile PBS, followed by centrifugation. The pellet was re-suspended in Pharma Lyse™ (BD Biosciences, 349202) red cell lysis buffer and spun at for 5 min. The pellets were re-suspended and pooled in 50 ml MACS buffer, and then, cells were counted using a Beckman Coulter Vi-CELL XR cell counter. Human anti-CD14 MicroBeads (Miltenyl Biotec) were mixed with cells at a dose of 1 ml per 5 × 10^8^ cells and placed at 4 ℃ to stain for 15 min. CD14-positive monocytes were separated using MidiMACS Separator kit (Miltenyi Biotec, 130-042-301) according to the manufacturer’s protocol. Collected cells were re-suspended in growing media containing 5% Human AB serum (Sigma, H4522) were incubated at 37 ℃ in 5% CO_2_ for 7 days. Experiments were also performed using fresh human CD14 + monocytes from Lonza Group (2W-400B) which were matured in endotoxin-free DMEM medium supplemented with 5% human AB serum for 7 days according to the manufacturer’s protocol. For co-culture experiments, macrophages were cultured with tumor cells in RPMI with 10% dialysed FCS.

### In vitro co-culture experiments

**Fig. 1:**
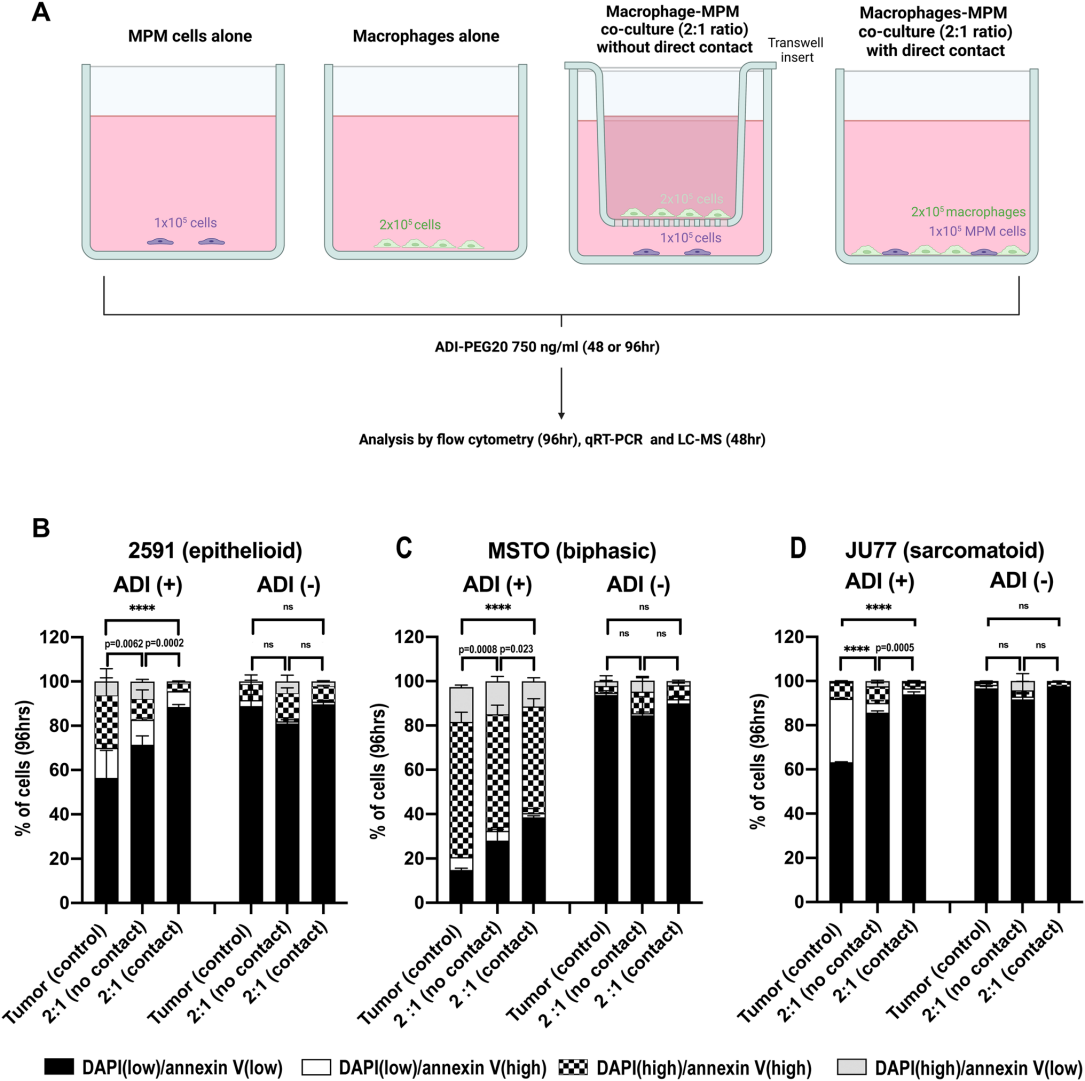
MPM cell lines are resistant to ADI-PEG20 upon co-culture with macrophages. Three ASS1-negative mesothelioma cell lines **A** 2591, **B** MSTO, and **C** JU77 were cultured alone and with macrophages, either with or without direct cell contact, and in the presence and absence of ADI-PEG20 (750 ng/ml). Two independent experiments were performed for each cell line in triplicate (*n* = 3). Viability was assessed at 4 days by flow cytometry. Bars show mean and standard deviation with statistical significance shown for the DAPI(low)/annexin V(low) cells only, ****p < 0.0001; Two-way ANOVA with post hoc Tukey’s test. Compared with the tumor (control) group, co-culture significantly increased MPM cell viability under ADI-PEG20: 2591 (F_3,48_ = 1481, df = 3); MSTO (F_3,48_ = 1193, df = 3); and JU77 F_5,24_ = 11,533, df = 3)

To assess tumor cell viability, 1 × 10^5^ MPM cells were seeded onto 6-well plates. At the same time, 2 × 10^5^ macrophages were plated alone or in co-culture with the MPM cells with or without direct cell contact (Fig. 1A). In the wells without direct cell contact, a 0.4 µm pore Transwell cell culture insert (BD Falcon, WZ-13009–26) was placed in each well. 2 × 10^5^ macrophages were then added on top of the insert for a ratio of 1:2 tumor cells to macrophages (Fig. 1A). After 24 h, the medium was discarded and the cells were gently washed three times with PBS. New medium was supplemented with ADI-PEG20 added at a concentration of 750 ng/ml. The plates were then incubated for 4 days. Following this, the cells were collected for flow cytometry staining. For qRT-PCR and western blot analysis, co-culture experiments were set up as described above but without direct cell contact. Cells were incubated for 48 h and then lysed with Buffer RLT lysis buffer (for qRT-PCR) or NP40 (for Western Blotting). For liquid chromatography–mass spectrometry, cells were incubated for 48 h. Following this, spun supernatant (100 µl) from tumor cells and macrophages co-cultured and individually cultured, together with control, was added to 300 µl of cold methanol and immediately vortexed for 5 min and put on ice. Finally, samples were spun down again and supernatants were transferred to a new Eppendorf tube, and then dried in a speed vac for 1 h, and dried extracts were stored at − 80 degrees for future analysis.

### Flow cytometric analysis

Tumor cells and macrophages were harvested with cold PBS using a cell scraper to gently detach the macrophages, and centrifuged at 1500RPM for 5 min, the supernatant was aspirated from each sample, and the cells were re-suspended in PBS. The washed cells were then re-centrifuged, the supernatant was discarded, and the cells (< 1 × 10^6^) were re-suspended in 100 µl annexin-binding buffer (Thermo Fisher Scientific, V13241). 5 µl of Alexa Fluor 488 annexin V (Thermo Fisher Scientific, V13241), 5 µl of CD14 (APC, BD Pharminogen, 555399), and 5 µl of CD11b (PE, BD Pharmingen, 550282) were added to each 100 µl cell suspension and the samples were incubated at 4 ºC for 30 min. After the incubation period, 400 µl of annexin-binding buffer was added to each sample and gently mixed, and the samples were then kept on ice for flow cytometry. Immediately prior to analysis, DAPI was added to each sample for assessment of dead cells. Apoptotic tumor cells were taken to be the percentage of Annexin V-positive cells. Viability was assessed only in the tumor cells after exclusion of macrophages using CD14 and CD11b staining.

### Argininosuccinate rescue

MPM (MSTO) tumor cells were subjected to a constant flow of RPMI media alone, with ADI-PEG20 or with ADI-PEG20 and argininosuccinate ((Sigma-Aldrich, 918149-29-8) for 24 h. 4 × 10^5^ cells were seeded onto m-Slide VI 0.4 IbiTreat and allowed to attach overnight. The slide was connected the following day to a peristaltic pump using sterile Tygon^®^ ND-100–65 Medical/Surgical Tubing (1/32″ 1/16 × 1/8″), providing a constant flow (100 ml/hr) of: a) full RPMI media (control); b) media supplemented with ADI-PEG20 (750 ng/ml); and c) media supplemented with ADI-PEG20 (750 ng/ml) together with argininosuccinate (0.5 µg/ml). After 24 h of incubation, tumor cells were stained with 4% Trypan Blue for 5 min and counted.

### Immunohistochemistry

ASS1 immunohistochemistry was performed as described previously [11]. Paraffin sections were dewaxed in xylene followed by incubation in 100% ethanol for 5 min. Endogenous peroxidase was blocked using 100% methanol and 3% hydrogen peroxide for 10 min. Antigens were retrieved in preheated 0.1 M citrate (pH 6) buffer for 10 min. Sections were blocked in horse serum (Thermo Fisher Scientific, 16,050,130). Primary antibody against ASS1 from Aviva System Biology (OACA09746) was applied for 1 h at 1:200 dilution followed by PBS washes and incubation with the secondary biotinylated antibody and incubation with horseradish peroxidase-conjugated avidin (ABC Standard: Vector Laboratories, PK-6100) and detection with 3,3′diaminobenzidine (DAB) chromogen (DakoCytomation, GV82511-2). Sections were also stained in Mayer’s Haematoxylin and Eosin (H&E). Representative images (at least 6) were collected with microscope Olympus BX51 manufactured by Olympus and ProgRes C5 digital camera and ProgRes Mac CapturePro 2.7.6 software manufactured by Jenoptic.

### RNA and bioinformatics analysis

RNA was obtained from the malignant mesothelioma cell line panel (2591, MSTO, JU77, and JU77 ASS1) at 24 h in the presence or absence of ADI-PEG20 (Polaris Pharmaceuticals, Inc., San Diego, California), and profiled using the Affymetrix GeneChip^®^ 3’IVT Human Genome U133 Plus 2.0 Array. The experiment was performed following the manufacturer’s protocol downloaded from the Affymetrix website: http://www.affymetrix.com/support/downloads/manuals/3_ivt_express_kit_manual.pdf. Quality control and differential expression analysis were performed within the open source R statistical environment (www.r-project.org) using the Bioconductor (http://www.bioconductor.org/) packages. After background correction and normalization, a linear model was fitted to the data using Limma [25]. Differentially expressed genes were determined by applying a double threshold of false discovery rate (FDR) (0.05) and fold change (at least 2). Altered expression patterns and biologically related pathways or networks were explored using IPA (Ingenuity Systems, www.ingenuity.com) and Metacore (GeneGo Inc.) with subsequent validation of candidate genes by quantitative real-time RT-PCR. Q-PCR analyses were performed using the ABI Prism 7500 Sequence Detection system Instrument and software (PE Applied Biosystems). RT-PCR was performed using sample cDNA (FAM), an internal control 18sRNA (VIC), and specific TaqMan^®^ probes for IL-1α, IL-8, CXCL2, CXCL3, and VEGF-A.

### ELISA analysis

IL1-α, IL-8, and VEGF-A in cell supernatant were analyzed using Quantikine ELISA kits (R&D Systems; DLA50, D8000C, DVE00). Human GRO-beta and GRO-gamma ELISA Construction kits (Antigenix America Inc.; RHF810CK, RHF820CKP) were used to measure the supernatant and plasma levels of CXCL2 and CXCL3, respectively. IL-1α, IL-8, and VEGF-A in human plasma were analyzed using custom V-PLEX Assays (Meso Scale Discovery, Maryland, USA; K151RBD-2, K151RAG-1, K151RHD-1). Patient blood samples were obtained after multicenter ethics approval (09/H1102/107 and 14/YH/0090) as described previously [16, 22]. Blood was centrifuged within 1 h of collection at 2500 rpm in a lithium-heparin containing tube; the plasma was then removed, aliquoted, and stored in a − 70** °**C freezer prior to thawing for ELISA analysis. ELISAs were carried out according to the manufacturer’s instructions.

### SiRNA transfection

The ASL (argininosuccinate lyase) siRNA smartpool was custom-made from Dharmacon and control (Control, D-001820-01) siRNA were used according to the manufacturer’s protocol (2 × 10^4^ tumor cells at 30% confluency in 6-well plates). Once knockdown was confirmed by qPCR, the knockdown was repeated and macrophages were added as per the previous co-culture experiments. After 4 days of incubation in ADI-PEG20, the cells were collected and cell viability was analyzed by flow cytometry. Apoptotic cells were taken to be the percentage of Annexin V-positive cells.

### Mass spectrometry (UPLC-MS/MS)

100 µl culture medium samples were extracted with 300 µl ice-cold methanol containing the internal standards (^13^C_6_ L-arginine and D4 L-citrulline). After centrifugation, methanolic extracts were evaporated to dryness. Then, dried extracts were reconstituted in 85% acidified acetonitrile and injected into the UPLC-MS/MS system. Analytes were resolved using an Accela UPLC (Thermo Scientific, UK) equipped with 1.7 µm HILIC Kinetex 2.1 × 50 mm UPLC column (Phenomenex, UK) and a mobile phase gradient of buffer A (water + 0.1% formic acid) and buffer B (acetonitrile + 0.1 formic acid) at a flow rate of 250 µl/min. Eluting compounds of interest were detected using a TSQ Vantage mass spectrometry system (Thermo Scientific, UK) in positive-ion mode. The optimum transitional daughter ions mass of each analyst was as follows: argininosuccinate m/z 291.1 → 70.2, arginine m/z 175.1 → 70.2, citrulline m/z 176.1 → 70.2, ^13^C_6_ arginine m/z 181.0 → 74.2, and D4 citrulline m/z 180.2 → 74.2. Plasma argininosuccinate was measured using untargeted metabolomics assay as described previously [26].

### Animal studies

Five-week-old female CD-1 homozygous Nu/Nu mice were purchased from Charles River Laboratories for xenograft studies. All mice were housed with a maximum of 6 mice per cage in a temperature-controlled, pathogen-free animal facility. Water and food were freely available. All experiments were commenced at age 6 weeks. Experiments were performed in accordance with the Home Office Animals (Scientific Procedures) Act in 1986 under animal license 70–7263 (10th July 2011) and with local welfare and ethical board approval. Subcutaneous xenografts were seeded into the right flank with 100 µl volume containing 3 × 10^6^ MSTO cells. Once the tumors were palpable (approximately 5–6 mm in diameter), treatment was initiated. Mice were placed into five different groups with 12 mice per group as follows: PBS (100 µl); CLIP (200 µl); PLIP (200 µl); ADI-PEG20 (100 µl at a dose of 5 IU/100 µl); ADI-PEG20 + CLIP. ADI-PEG20 (ADI) was obtained from Polaris Pharmaceuticals, Inc. (San Diego, CA, USA). Liposome preparations were obtained from Dr Nico van Rooijen, Department of Molecular Cell Biology, Vrije Universiteit, Amsterdam. Liposomes contained either clodronate at 7 mg/ml (CLIP), or PBS as a vehicle control, manufactured as previously described [27]. Animals were injected into the peritoneum with 200 µl of CLIP or vehicle control. Intraperitoneal injections were performed into the mouse peritoneum using a 1 ml capacity syringe and a 25-gauge needle (BD microlance). 200 µl of CLIP (or PLIP as vehicle control) was injected twice a week for the first 3 doses to initiate macrophage depletion (Friday, Monday, and Wednesday), and then continued on a weekly basis (every Wednesday) for the duration of the experiment for maintenance. 100 µl of ADI PEG20 (or PBS as vehicle control) was injected on a weekly basis (every Thursday) after the first 3 doses of CLIP (or PLIP) had been administered. Mesothelioma tumor progression assessment was performed with the observer blinded to the therapy received by each group. Assessment of tumor volume initially involved measurement of the long axis and perpendicular axis of the tumors using 0–200 mm electronic digital calipers with 0.01 mm resolution. Tumor volumes were then calculated according to the following formula: tumor volume = ($\uppi$ w^2^l)/6. w = short axis; l = long axis. Tumors were measured twice-weekly.

### Reagents

Antibodies were obtained from the following sources: mouse anti-human ASS1 monoclonal antibody from BD Pharmingen and Cell Signaling Technology, Inc; rabbit anti-human ASS1 polyclonal antibody from Aviva Systems Biology; rabbit anti-human ASL polyclonal antibody from Atlas (UK); rat anti-mouse F4/80 monoclonal antibody from Abcam; anti-mouse AlexaFluor 488 and anti-rabbit AlexaFluor 568 from Invitrogen. Argininosuccinic acid, arginine (1119-34-2), and citrulline (C7629) were purchased from Sigma-Aldrich, UK, and ^13^C_6_ arginine and D4 citrulline were from Cambridge Isotopes laboratory, MA, USA. LC–MS grade water, acetonitrile, and formic acid were obtained from Fisher Scientific, UK.

### Statistical analysis

All statistical analysis was undertaken in GraphPad Prism (version 7 and 8). Three independent experiments were performed unless indicated. Experiments with greater than two groups were analyzed by 1-way ANOVA or 2-way ANOVA, depending on the number of variables. For missing values, the mixed-effects model was used. Experiments to determine the difference in plasma cytokine levels in one group before and after ADI-PEG20 administration were calculated using the non-parametric Wilcoxon signed-rank test. A *p* value of < 0.05 was considered to be statistically significant.

## Results

### ASS1-negative MPM cell and macrophage co-culture under ADI-PEG20 treatment

We used an in vitro co-culture cell model (Fig. 1A) to investigate the increased tumoral infiltration of CD68^pos^ ASS1^pos^ macrophages, that was identified in patients with ASS1-negative mesothelioma progressing on pegargiminase-based therapy in the aforementioned TRAP study [22]. Human PBMC-derived macrophages were co-cultured with three histologically distinct ASS1-negative wild-type MPM cell lines: 2591 (Fig. 1B) MSTO (Fig. 1C) and JU77 (Fig. 1D), with and without direct cell contact, and in the presence and absence of ADI-PEG20. Tumor cell were protected from ADI-PEG20 cytotoxicity by up to 30%, as assessed by an increase in low DAPI/low Annexin V labeling, when MPM cells were co-cultured with macrophages, compared with tumor cells cultured alone. Moreover, the effect on tumor cell viability was evident with and without direct macrophage contact, implying a soluble resistance factor.

### Regulation of pro-inflammatory cytokines in ASS1-negative MPM cells by ADI-PEG20

**Fig. 2:**
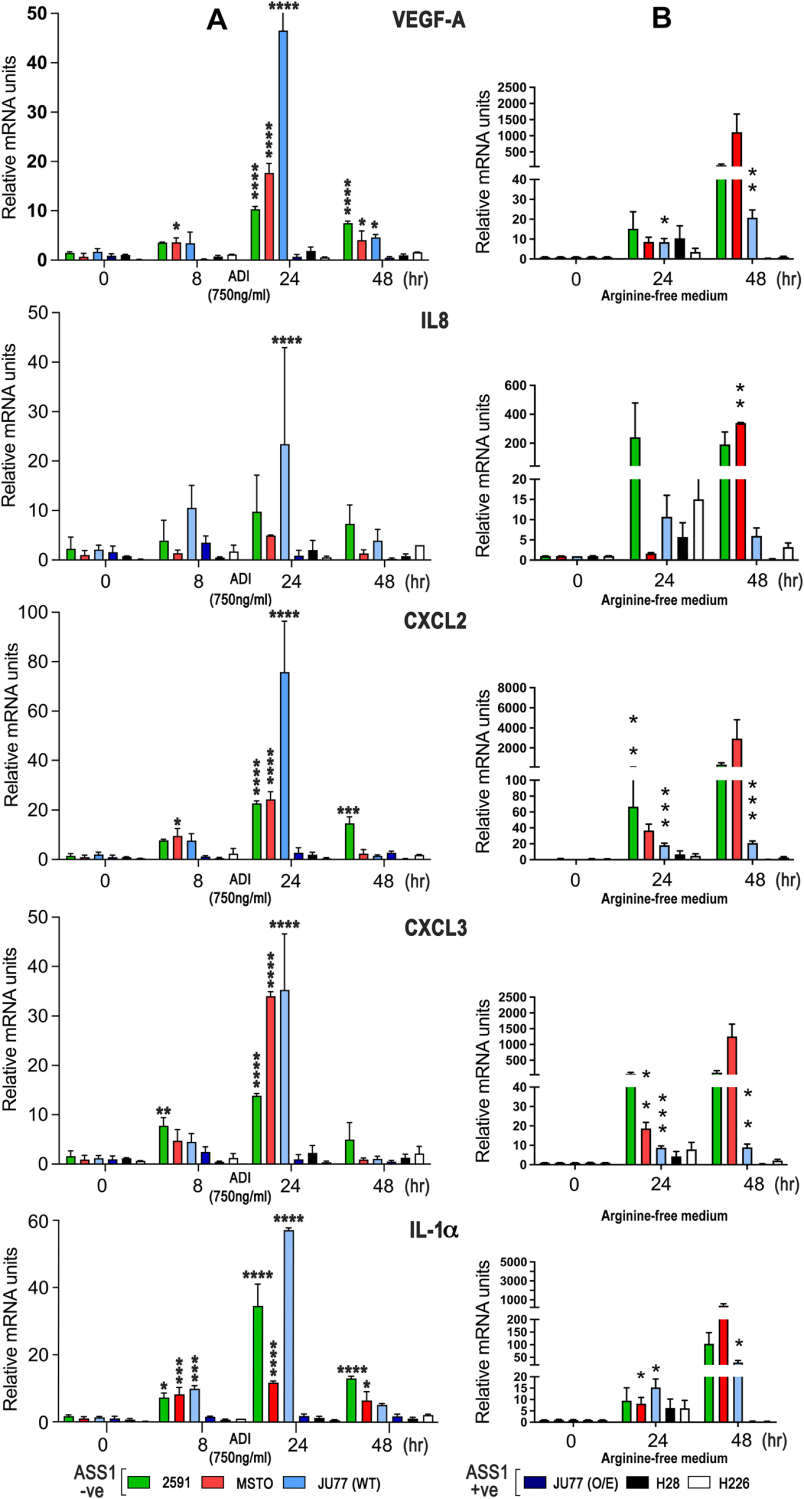
qPCR assay validation of ADI-PEG20-induced MPM cytokine gene signature. **A** Three ASS1 negative and three ASS1-positive control mesothelioma cell lines were analyzed for induction of cytokine mRNA following ADI-PEG20 treatment at 0, 8, 24, and 48 h: VEGF-A, IL-8, CXCL2, CXCL3, and IL-1α. Experiments were performed in triplicate for each cell line (*n* = 3), with bars representing the mean and standard deviation: *p < 0.05, **p < 0.01, ***p < 0.001, ****p < 0.0001; Two-way ANOVA with post hoc Tukey’s test. Compared with unstimulated levels, ADI-PEG20 significantly increased cytokine levels in the three ASS1-negative MPM cell lines: VEGF-A (F_5,45_ = 135.8, df = 15; *p* < 0.0001); IL-8 (F_15,42_ = 1.912, df = 15; *p* = 0.05); CXCL2 (F_15,41_ = 37.82, df = 15; *p* < 0.0001); CXCL3 (F_15,40_ = 31.94, df = 15; *p* < 0.0001); and IL-1α (F_15,40_ = 62.19, df = 15; *p* < 0.0001). **B** Similar results were obtained across the ASS1-negative and ASS1-positive MPM cell lines using arginine-free media: mixed-effects model, *p < 0.05, **p < 0.01, ***p < 0.001; VEGF-A (F_8,72_ = 11.69, *p* < 0.0001); IL-8 (F_8,45_ = 2.604, df = 1; *p* = 0.0197); CXCL2 (F_8,50_ = 2.384, df = 8; *p* < 0.0293); CXCL3 (F_8,48_ = 31.17.80, df = 1; *p* < 0.0001); and IL-1α (F_8,48_ = 6.831, df = 1; *p* < 0.0001)

**Fig. 3:**
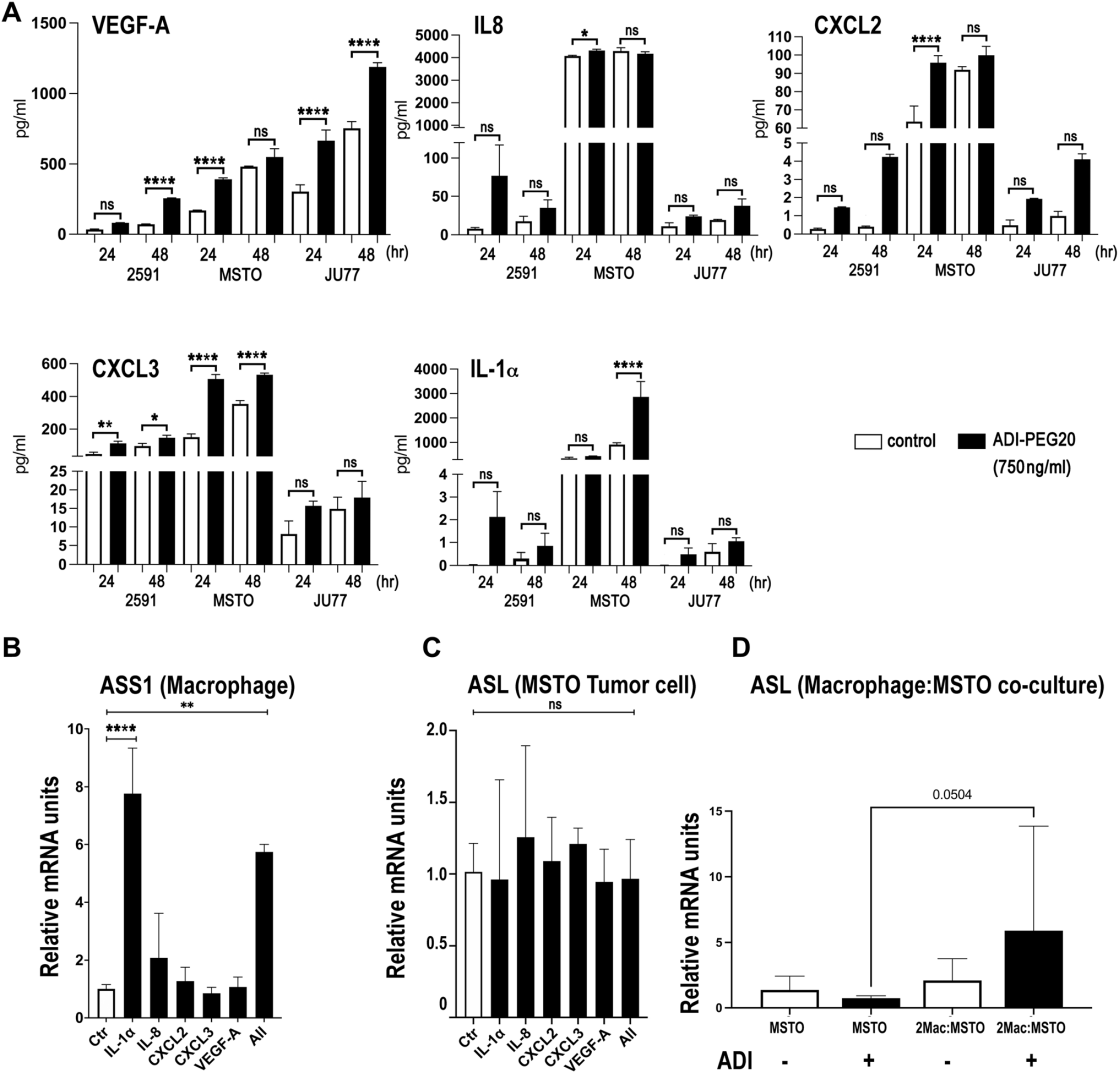
MPM cytokine induction by ADI-PEG20 and co-ordinate UC enzyme regulation. **A** Release of the pro-inflammatory cytokines VEGF-A, IL-8, CXCL2, CXCL3, and IL-1α in the cell supernatant of the ASS1-negative MPM cell lines was detected by ELISA at 24 and 48 h following treatment with ADI-PEG20 (*n* = 3). ELISAs were run in duplicate, with values representing the mean and standard deviation: *p < 0.05, **p < 0.01, ****p < 0.0001; Two-way ANOVA with post hoc Tukey’s test. **B** Macrophage ASS1 mRNA and **C** MSTO tumoral cell line ASL mRNA expression following stimulation by IL-1α (2 ng/ml), IL-8 (2 ng/ml), CXCL2 (0.1 ng/ml), CXCL3 (0.5 ng/ml), and VEGF-A (1 ng/ml) individually or in combination using concentrations detected by ELISA above (*n* = 6), as assessed using qPCR with GAPDH mRNA for normalization. Values represent the mean and standard deviation of the mean: **p < 0.01, ***p < 0.001; One-way ANOVA with post hoc Holm–Sidak test as indicated. **D** ASL mRNA expression in the MSTO tumor cell line following co-culture with macrophages ± ADI-PEG20 (750 ng/ml). Values represent the mean and standard deviation: *p* < 0.05 (*n* = 9); two-way ANOVA with post hoc Tukey’s test

To identify which tumor-dependent pathways related to macrophage signaling, we analyzed the gene expression profile of the three ASS1-negative MPM cell lines (2591, MSTO and JU77) treated with ADI-PEG20. Among the most highly up-regulated genes identified in the ASS1-negative MPM cell lines by 24 h of ADI-PEG20 treatment were the pro-inflammatory interleukin IL-1α, the CXCR-2 dependent chemoattractant cytokines IL-8 (CXCL8), CXCL2, CXCL3, and vascular endothelial growth factor-A (VEGF-A), whereas JU77 cells overexpressing ASS1—selected as a suitable control for ADI-PEG20 resistance—displayed minimal fold-change values for cytokine gene expression (Table 1). We validated that the cytokine gene expression signature following ADI-PEG20 treatment was specific to ASS1-negative MPM cells using qPCR with a 5- to 70-fold increase of cytokine mRNA peaking by 24 h (Fig. 2A) and confirmed that arginine-deficient medium also increased cytokine mRNA but by 5- to 3000-fold and peaking by 48 h (Fig. 2B). Overall, ADI-PEG20 induced detectable levels of soluble cytokines and chemokines in the cell supernatant of the ASS1-deficient MPM cell lines, particularly the MSTO biphasic cell line (Fig. 3A), but this was not always significant compared to controls, when measured at the lower levels of detectability by ELISA. To determine the relevance of the pro-inflammatory cytokine profile to the tumor cell-macrophage metabolic cooperation, we studied whether the tumor-derived pro-inflammatory cytokines induced by ADI-PEG20 modulated ASS1 and ASL expression in macrophages and tumor cells, respectively. IL-1α alone increased ASS1 mRNA expression in macrophages (Fig. 3B), while tumoral ASL expression was unaffected by cytokine stimulation (Fig. 3C). Nonetheless, ASL mRNA increased in MSTO tumor cells upon co-culture with macrophages indicating co-ordinate regulation of urea cycle enzymes with ADI-PEG20 treatment (Fig. 3D).

**Table 1:** Affymetrix high density oligonucleotide expression array analysis

GENE*	2591	MSTO	Ju77 (wild-type)	Ju77 ASS1 + ve
VEGFA	4.03	4.17	3.33	0.37
IL-1α	4.29	4.44	2.22	0.26
CXCL2	7.77	2.58	7.45	0.51
CXCL3	3.38	5.00	4.47	0.52
IL-8	3.82	3.48	4.11	0.66

Gene expression data detailing the fold change in pro-inflammatory cytokines following ADI-PEG20 treatment by 24 h in the ASS1-negative MPM cell line panel and using an ASS1-positive MPM cell line control. The complete gene expression dataset is accessible (E-MTAB-12873) via the Gene Expression Omnibus (GEO): https://www.ncbi.nlm.nih.gov/geo/

*Fold change in most highly up-regulated pro-inflammatory cytokines with ADI-PEG20 treatment (750 ng/ml) in a panel of MPM cell lines analyzed using the Affymetrix U133 plus 2.0 microarray platform. ASS1-Ju77 as an overexpressing cell line control

### Macrophages secrete argininosuccinate bypassing ADI-PEG20-induced cytotoxicity

**Fig. 4:**
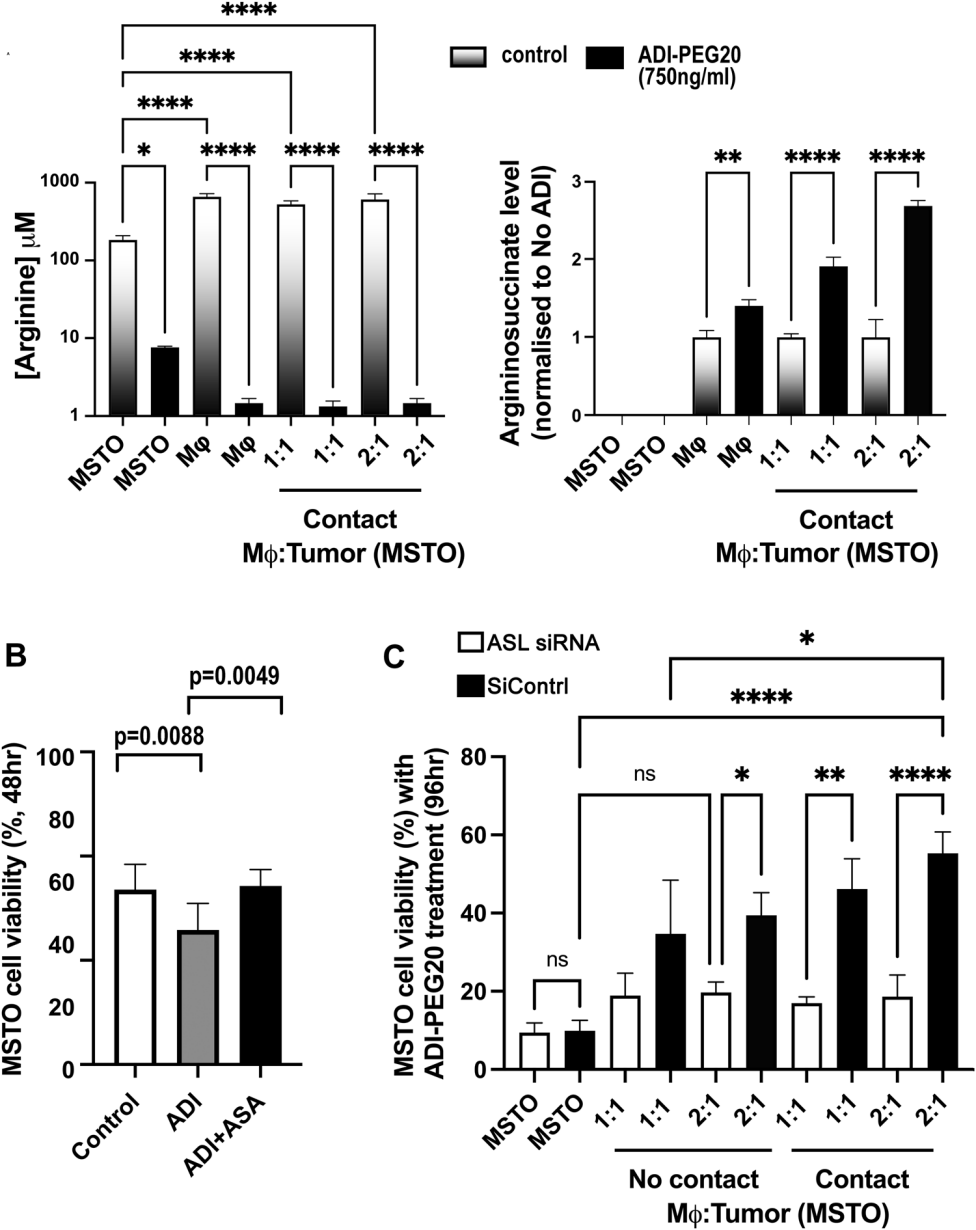
Macrophage-derived argininosuccinate bypasses ADI-PEG20 cytotoxicity in MPM cells. **A** Mean [arginine] and [argininosuccinate] in the supernatant from macrophage and MSTO tumor cells alone and co-cultured (with and without direct cell contact) by 48 h following ADI-PEG20 treatment using LC/MS (*n* = 3). Bars show mean and standard deviation: *p < 0.05, **p < 0.01, ****p < 0.0001; two-way ANOVA with post hoc Tukey’s test ([arginine], F_7,14_ = 92.90, df = 7; *p* < 0.0001), [argininosuccinate], (F_7,14_ = 213.3, df = 7; *p* < 0.0001); (**B** Argininosuccinate rescue (using 0.5 µg/ml, i.e., peak concentration measured above) with MSTO tumor cell viability assessed at 2 days in the presence of ADI-PEG20 (n = 6). Bars represent mean values and standard deviation: **p < 0.01; one-way ANOVA with uncorrected Fisher’s LSD post hoc test (F_2,15_ = 6.670, df = 2; *p* < 0.0085); and **C** the effect of ASL mRNA knockdown in the MSTO MPM cell line on the metabolic resistance conferred by macrophage-derived argininosuccinate. Cell viability was assessed at 4 days by flow cytometry. Bar values representing the mean and standard deviation (*n* = 3): *p < 0.05, **p < 0.01, ****p < 0.0001; two-way ANOVA with post hoc Tukey’s test (F_9,18_ = 17.30, df = 9; p < 0.0001)

Given the induction of pro-inflammatory cytokines with the ability to both attract macrophages and activate the urea cycle pathway in co-cultured macrophages and tumor cells, we hypothesized that argininosuccinate, the immediate precursor for arginine synthesis by ASL, may be the critical resistance factor to macrophage-mediated resistance to ADI-PEG20 therapy. Therefore, we analyzed the levels of arginine and argininosuccinate in the supernatant from tumor cells and macrophages, alone and in co-culture, with and without ADI-PEG20, by liquid chromatography–mass spectrometry. As expected, the arginine concentration decreased to negligible levels after 48 h of ADI-PEG20 treatment in all treatment groups (Fig. 4A), while there was no detectable argininosuccinate in MSTO cells cultured alone. In contrast, the argininosuccinate concentration in cell supernatant from macrophages cultured alone increased measurably with ADI-PEG20. Conversely, upon co-culture of macrophages with MPM cells, we observed a relative doubling of argininosuccinate in the cell supernatant by 48 h of ADI-PEG20 treatment, further indicating that macrophages secrete this amino acid. Furthermore, 0.5 μg/ml of argininosuccinate rescued ASS1-negative MPM cells from ADI-PEG20 cytotoxicity (Fig. 4B). To confirm the role of argininosuccinate in macrophage-mediated resistance, we transfected the MSTO MPM cell line with siRNA directed against ASL and co-cultured these cells with macrophages in the presence of ADI-PEG20. We found that silencing tumoral ASL mRNA abrogated the macrophage-mediated resistance to ADI-PEG20 (Fig. 4C).

### Pro-inflammatory cytokines and argininosuccinate linked to clinical MPM progression

**Fig. 5:**
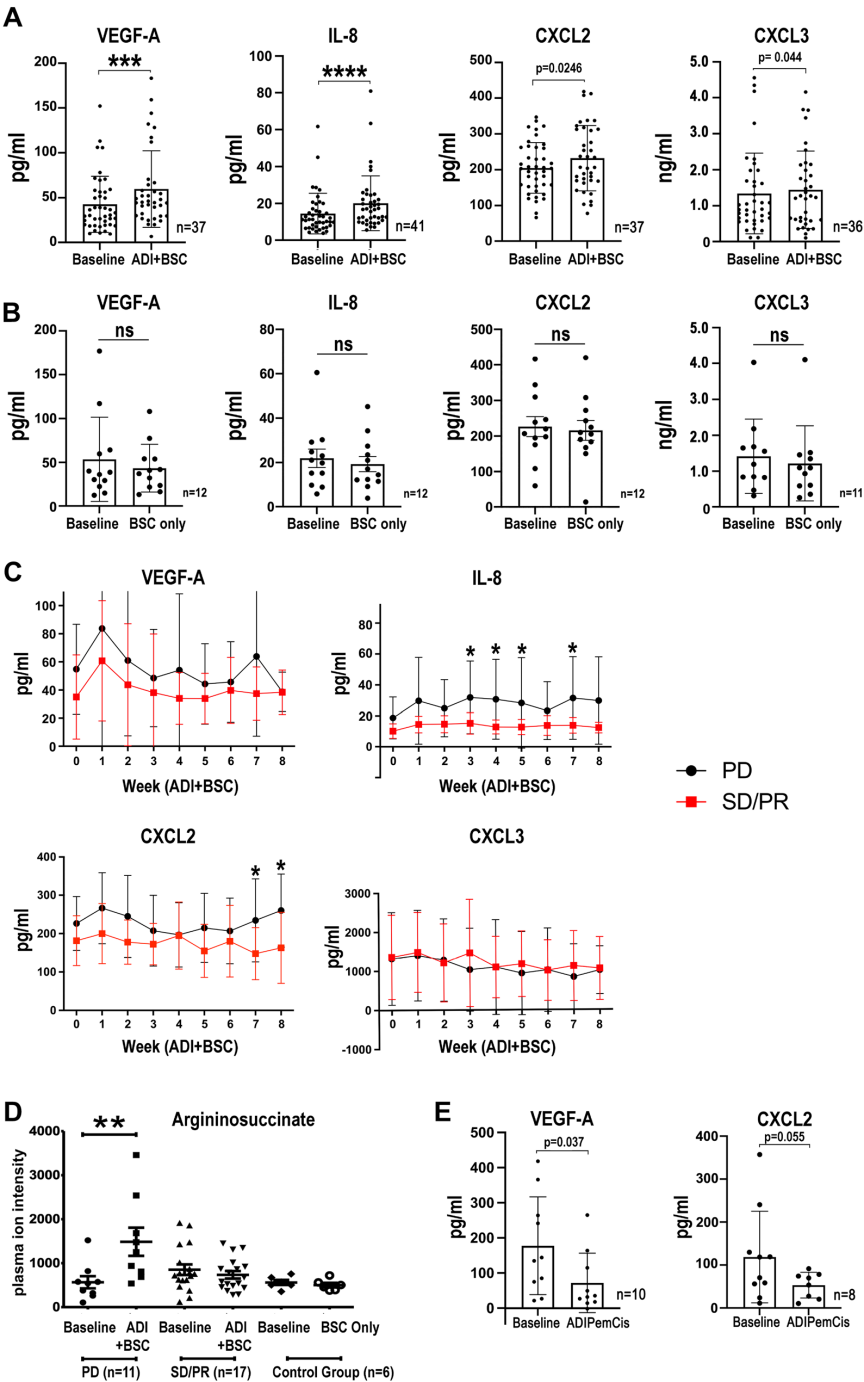
Clinical validation of cytokine signature and argininosuccinate.** A** Plasma VEGF-A, IL-8, CXCL2, and CXCL3 concentrations of patients on the ADAM study randomized to ADI-PEG20 and BSC compared with baseline levels; and **B** control patients randomized to BSC alone compared to baseline levels (IL-1α not detected in either group). Bar values represent mean and standard deviation: *p < 0.05, ***p < 0.001, ****p < 0.0001; Wilcoxon signed-rank test. **C** Plasma cytokine concentrations in patients whose disease had progressive disease (PD) compared with patients who had stable disease/partial response (SD/PR) at the 8 week assessment by CT imaging were analyzed in the ADI-PEG20 plus BSC treatment group. Data represent mean values with standard deviation: *p < 0.05; two-way ANOVA followed by Sidak’s multiple comparison’s test, VEGF-A (F_1,317_ = 12.25, df = 1; *p* < 0.0005), IL-8 (F_1,318_ = 56.46, df = 1; *p* < 0.0001), CXCL2 (F_1,295_ = 31.0, df = 1; *p* < 0.0001), and CXCL3 (F_1,281_ = 0.9333, df = 1; *p* = 0.335). **D** Argininosuccinate was detected in plasma from all patients and was only significantly increased in the ASS1-deficient patients with disease progression by week 8 of ADI-PEG20 plus BSC treatment, whereas levels were unchanged in patients displaying SD/PR as analyzed by CT imaging. **p < 0.01; by two-way ANOVA. **E** Plasma VEGF-A and CXCL2 concentrations of patients receiving ADI-PEG20 plus chemotherapy compared with baseline levels within the first 2 months on the TRAP study. Bar values represent mean and standard deviation: *p < 0.05; Wilcoxon signed-rank test

**Fig. 6:**
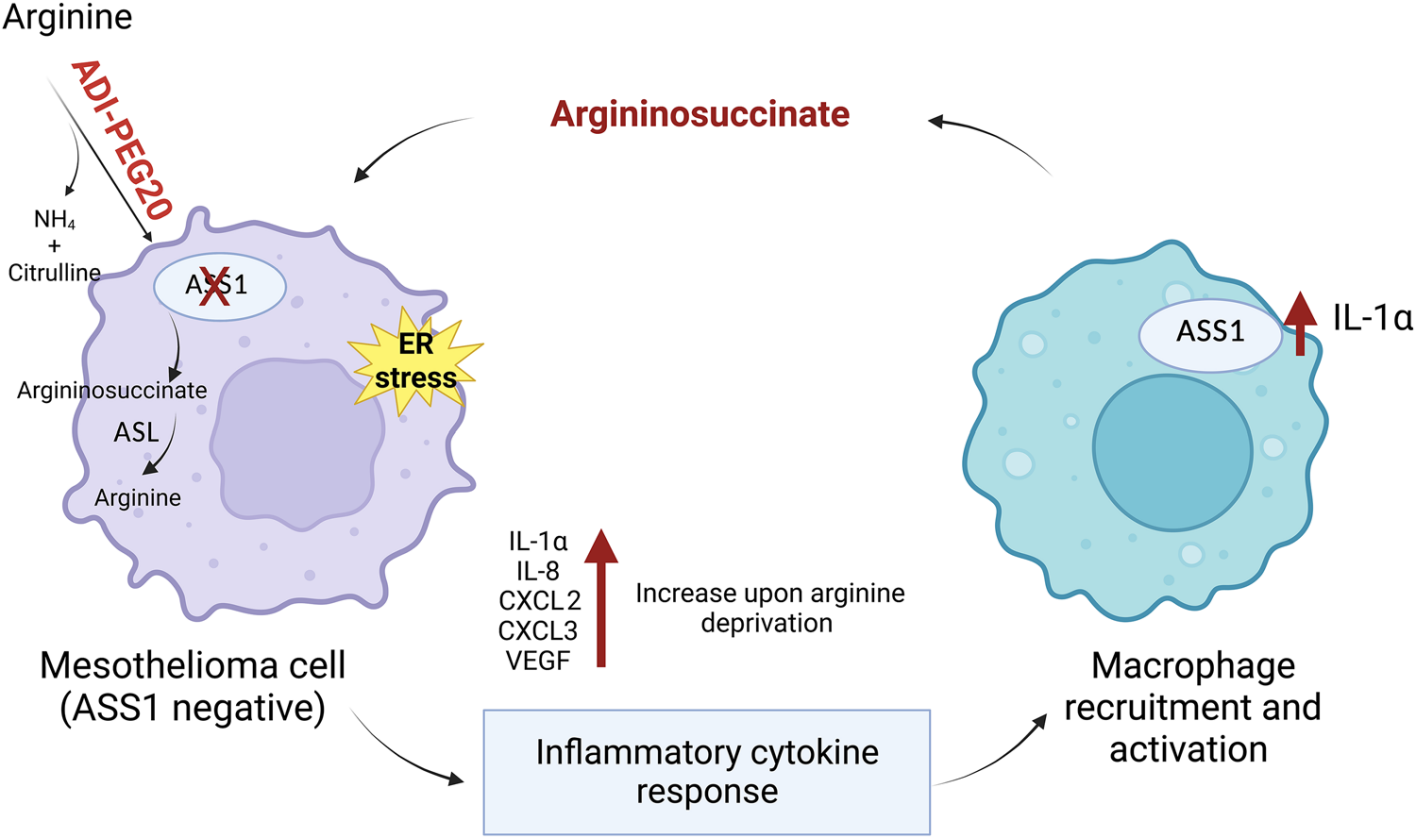
Graphical abstract displaying cytokine-driven macrophage-mediated resistance mechanism to ADI-PEG20 therapy in ASS1-deficient mesothelioma

To corroborate the preclinical cytokine data from the human MPM cell lines, we analyzed blood samples from patients with ASS1-deficient MPM treated with ADI-PEG20 plus Best Supportive Care (BSC; analgesia and steroids permitted only) or BSC only (control group) in the ADAM trial (*n* = 68) [10]. ADI-PEG20 plus BSC induced a significant increase in plasma VEGF-A, IL-8, CXCL2, and CXCL3 in patients with ASS1-deficient tumors (Fig. 5A) whereas no change was detected in the BSC only group (Fig. 5B); in contrast, IL-1α was undetectable in patients on ADI-PEG20 treatment and the BSC only group. Moreover, patients on ADI-PEG20 monotherapy with early disease progression by computerized tomography (CT) imaging had significantly higher levels of VEGF-A, IL-8, and CXCL2 compared with those who demonstrated disease control with arginine deprivation (Fig. 5C). Likewise, in the early metabolic progressors, we detected a significant increase in plasma argininosuccinate levels following ADI-PEG20 treatment, with no change detected in patients demonstrating disease control (Fig. 5D). Finally, limited analysis of plasma from patients treated with ADI-PEG-20 plus chemotherapy (cisplatin and pemetrexed) on the TRAP study indicates that chemotherapy may initially suppress the pro-inflammatory response seen with ADI-PEG20 monotherapy (Fig. 5E). Taken together, our results support a role for ASS1-expressing TAMs under cytokine control mediating resistance to arginine deprivation via argininosuccinate as a critical metabolite fuelling mesothelioma growth (Fig. 6).

### Macrophage depletion and ADI-PEG20 suppresses tumor growth in vivo

**Fig. 7:**
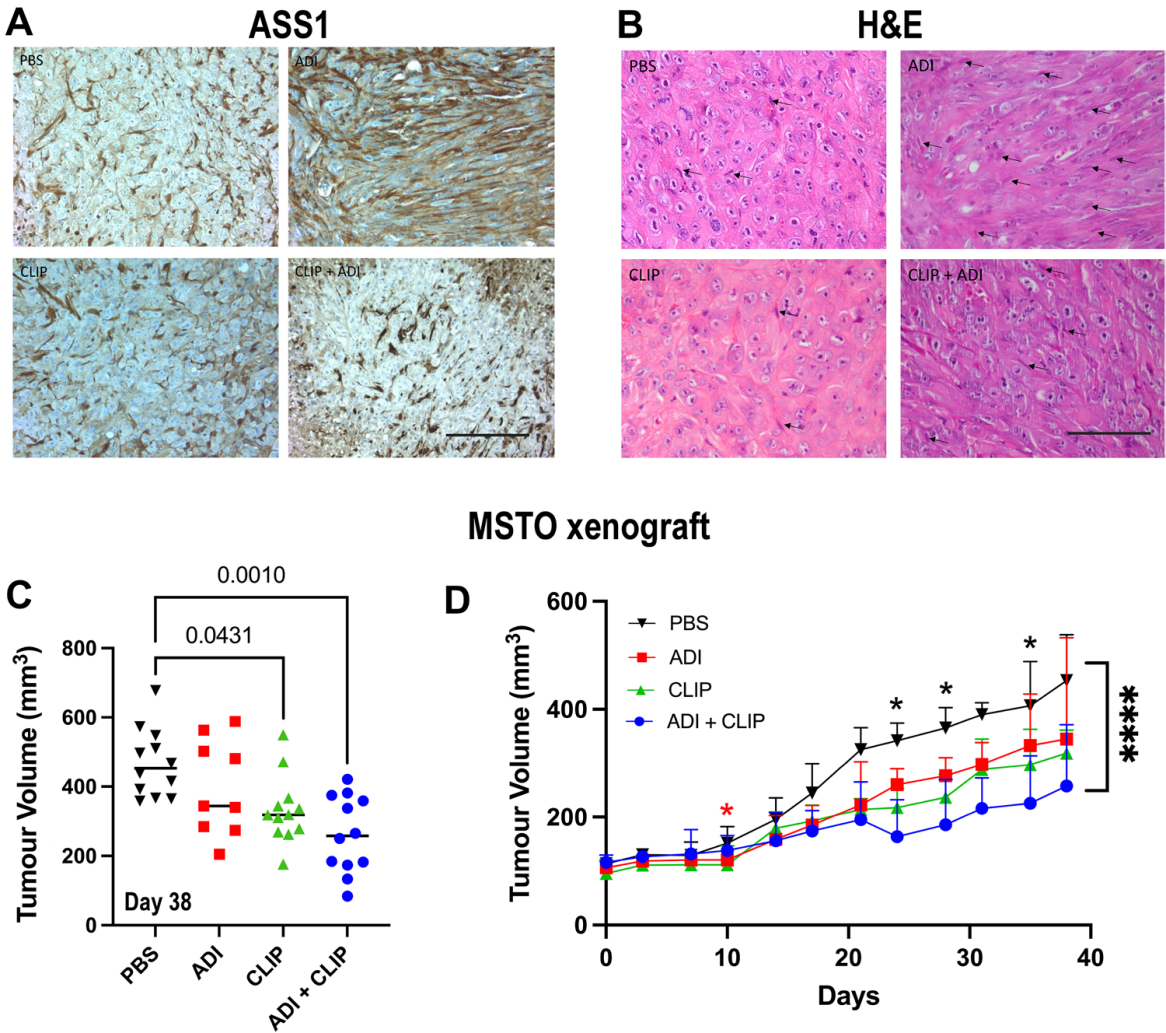
MSTO murine xenograft model. **A** ASS1 immunohistochemistry (X400; 100 mM scale bar) performed in paraffin sections of MSTO tumors developing in CD-1 mice treated with PBS, ADI-PEG20, CLIP, or CLIP + ADI-PEG20. **B** Corresponding H&E sections (X400; 100 µm scale bar) showing macrophages adjacent to tumor cells (elongated cells—arrowed) in the MSTO mesothelioma xenograft model. **C** Final MSTO tumor volumes (*n* = 12 per treatment group) with statistical significance analyzed by two-way ANOVA and the post hoc Tukey’s test. **D** MSTO tumor growth (38 day study with a prior pilot experiment): *p < 0.05, *p < 0.0001; two-way ANOVA with post hoc Tukey’s multiple comparisons test

Since macrophages rescued ASS1-negative MPM cells from ADI-PEG20-induced cytotoxicity via argininosuccinate, we employed clodronate-containing liposomes (CLIP) which deplete macrophages in mesothelioma mouse models, in combination with arginine deprivation in vivo [28]. Thus, human MSTO cells implanted subcutaneously in athymic mice were treated with PBS, CLIP, ADI-PEG20, or a combination of ADI-PEG20 and CLIP. There was no tumoral re-expression of ASS1, a known mechanism of resistance to ADI-PEG20, but ASS1 was detected in infiltrating stromal cells consistent with macrophages (Fig. 7A and B). CLIP therapy reduced ADI-PEG20-directed infiltration of MSTO tumors by macrophages and suppressed tumor growth more robustly compared to CLIP alone (Fig. 7B and C). Moreover, ADI-PEG20 which displayed limited single-agent activity in the MSTO xenograft model by day 10, was additive with CLIP when compared to ADI-PEG20 alone at subsequent timepoints providing in vivo evidence for a key stromal role in mediating resistance to arginine deprivation (Fig. 7D).

## Discussion

Our study addresses a novel stromal-mediated resistance mechanism to pegargiminase in patients with mesothelioma that links a tumor-derived pro-inflammatory cytokine network to the recruitment of macrophages, that feed arginine-depleted MPM cells with argininosuccinate. Moreover, depleting macrophages potentiated the effect of arginine suppression in our in vivo murine tumor model, emphasizing an important role for stromal modulation in combination with arginine deprivation for mesothelioma and related arginine-auxotrophic cancers.

Aberrant tumor metabolism is recognized as one of the key hallmarks of cancer with metabolic symbiosis within tumors an emerging field of study, in which cancer and stromal cells including macrophages cooperate to promote proliferation, invasion, metastasis, and therapeutic resistance [29–38]. Here, we show that metabolite crosstalk between tumor cells and macrophages is a key survival strategy in arginine-auxotrophic mesothelioma treated with ADI-PEG20. This parallels studies where co-cultured fibroblasts deficient in either ASS1 or ASL retained viability via the synthesis and uptake of argininosuccinate [39]. We demonstrated the co-ordinate up-regulation of ASS1 and ASL implicated in the macrophage-mediated resistance to ADI-PEG20 and that knockdown of tumoral ASL abrogated this resistance pathway. Several pro-inflammatory cytokines, including TNFα, IL-1β, and TGFβ, are known to induce up-regulation of ASS1 in endothelial, inflammatory, and malignant cells, in part via nuclear factor-kappa B [40, 41]. Here, we have identified IL-1α as a novel regulator of ASS1 expression in macrophages. However, we were unable to show that argininosuccinate is directly released from IL-1α-stimulated macrophages cultured alone, whereas a doubling in argininosuccinate was detected in the supernatant under co-culture and arginine-depleted conditions.

The ELR( +)CXC chemokines, together with VEGF-A, are also recognized chemotactic factors for macrophages [42, 43]. The increased tumoral macrophage infiltrate in patients progressing on ADI-PEG20 therapy was recapitulated in our human MPM xenograft studies. The induction of the pro-inflammatory cytokines by ADI-PEG20 is likely complex, involving activation of the ER stress response, feedback loops, and multiple downstream pathways, mediated by distinct transducers of specialized transcriptional programs, including spliced XBP1 [44–46]. In support of this, our bioinformatic analysis of ADI-PEG20 treated ASS1-negative mesothelioma cells demonstrated significant up-regulation of XBP1 in concert with the pro-inflammatory cytokine response.

It is noteworthy that our preclinical data are consistent with plasma analyses from patients with MPM on ADI-PEG20 monotherapy in the ADAM study. Increased IL-8, CXCL2, CXCL3, and VEGF-A were found with ADI-PEG20 monotherapy but not in the control arm (best supportive care only); IL-1α, however, was below the lower limit of detection of the assay. Furthermore, IL-8 and CXCL2 along with argininosuccinate were significantly elevated in the plasma of patients whose mesothelioma had progressed within the first 2 months of ADI-PEG20 therapy, compared with patients demonstrating disease control. IL-8, in particular, as a cytokine mediator of resistance to targeted anticancer agents has gained increasing prominence in recent years [47]. Interestingly, IL-8 was also identified as a potential mediator of resistance to glutamine deprivation in a recent preclinical model of osteosarcoma, involving ER stress and mTORC1-mediated activation of JNK signaling [48]. Therefore, the consistent increase in plasma IL-8 and argininosuccinate may have utility as early resistance biomarkers to ADI-PEG20. Further study of pro-inflammatory cytokines is warranted in clinical trials of arginine deprivation, including the recently completed phase 3 ATOMIC-meso study in patients with non-epithelioid mesothelioma, and significantly the first study to show an overall survival benefit for pegargiminase in cancer (NCT02709512) [49]. Moreover, regarding the increased VEGF-A response correlating with disease relapse on ADI-PEG20, there may be a role for depleting arginine in combination with anti-angiogenic strategies which have shown activity in appropriately selected patients with mesothelioma [50].

Notably, depleting macrophages within the mesothelioma microenvironment potentiated ADI-PEG20 activity in the human mesothelioma xenograft model. Liposomal clodronate (CLIP) is well established as a macrophage depleting agent in mouse models but is restricted clinically due to systemic toxicity related to pan-macrophage elimination [28]. Alternative therapeutic strategies include targeting CSF-1 or the CSF-1 receptor, and antagonizing CXCR2 [51, 52]. Recently, Chu et al. delineated activation of TREM1/CCL2 and AKT/mTOR/STAT3 as an additional mechanism to ASS1 re-expression, mediating ADI-PEG20 resistance in breast and prostate cancer cells [53]. In contrast, in the malignant mesothelioma cell line panel, CCL2 expression was downregulated compared to the marked increase in CXCR2-dependent chemokines, IL-1α, and VEGF-A underscoring the critical role played by cell type in the context of arginine deprivation therapy.

In summary, our data implicate a novel macrophage-mediated mechanism of resistance to ADI-PEG20 in ASS1-negative MPM, namely the delivery of argininosuccinate by the macrophage-rich tumor microenvironment under cytokine control. Further research is required to elucidate whether this metabolic relationship exists in the context of other ASS1-negative cancers and stromal cells. Ultimately, targeting macrophages alongside ADI-PEG20 therapy may potentially benefit other refractory cancers exhibiting a metabolic vulnerability for arginine.

## Limitations

Validation of a causal role for macrophage-derived ASS1 in mediating stromal resistance to ADI-PEG20 was not pursued in the co-culture model due to a lack of specific ASS1 antagonists. Further interrogation of the cytokine data is warranted in the co-culture model using specific cytokine and chemokine antagonists.

## Supplementary Information

Below is the link to the electronic supplementary material.

Supplementary file1 (TIF 488 KB)

Supplementary file2 (JPG 41828 KB)

## Data Availability

The datasets are available from the corresponding author upon reasonable request. Flow cytometry data and additional IHC images are included in Supplementary materials.
