# Correction: A role for macrophages under cytokine control in mediating resistance to ADI-PEG20 (pegargiminase) in ASS1-deficient mesothelioma

**DOI:** 10.1007/s43440-023-00487-z

**Published:** 2023-05-17

**Authors:** Melissa M. Phillips, Iuliia Pavlyk, Michael Allen, Essam Ghazaly, Rosalind Cutts, Josephine Carpentier, Joe Scott Berry, Callum Nattress, Shenghui Feng, Gunnel Hallden, Claude Chelala, John Bomalaski, Jeremy Steele, Michael Sheaff, Frances Balkwill, Peter W. Szlosarek

**Affiliations:** 1 Center for Cancer Biomarkers and Biotherapeutics, Barts Cancer Institute (BCI)-a Cancer Research UK Center of Excellence, Queen Mary University of London, John Vane Science Center, London, EC1M 6BQ UK; 2 Department of Medical Oncology, Barts Health NHS Trust, St Bartholomew’s Hospital, West Smithfield, London, EC1A 7BE UK; 3 Center for Tumor Microenvironment, Barts Cancer Institute (BCI)-a Cancer Research UK Center of Excellence, Queen Mary University of London, John Vane Science Center, London, EC1M 6BQ UK; 4 Centre for Haemato-Oncology, Barts Cancer Institute (BCI)-a Cancer Research UK Center of Excellence, Queen Mary University of London, John Vane Science Center, London, EC1M 6BQ UK; 5 Medicines and Healthcare Products Regulatory Agency (MHRA), London, UK; 6 Breast Cancer Now Toby Robins Research Centre, The Institute of Cancer Research, London, UK; 7 Polaris Pharmaceuticals, Inc., San Diego, CA 92121 USA; 8 Department of Histopathology, Barts Health NHS Trust, Royal London Hospital, London, E1 1BB UK

**Correction to: Pharmacological Reports (2023)** 10.1007/s43440-023-00480-6

In this article following figures had incorrect units.

1. Figure 3A panel legend has the incorrect units for ADIPEG20 (“mg/ml” should be “ng/ml”)
2. Figure 3D legend says incorrectly “75ong/ml” but should be “750 ng/ml”
3. Figure 4B legend has the incorrect units for argininosuccinate (“mg/ml” but “µg/ml”)
4. Figure 7B legend has the incorrect units for the scale bar (“mM” but needs to be “µm”)

The original article has been corrected.

