# Supplementary figures and images for: A role for macrophages under cytokine control in mediating resistance to ADI-PEG20 (pegargiminase) in ASS1-deficient mesothelioma
Source: Pharmacol Rep. 2023 Apr 3;75(3):570–84. doi: 10.1007/s43440-023-00480-6 (PMC10227144; doi:10.1007/s43440-023-00480-6)

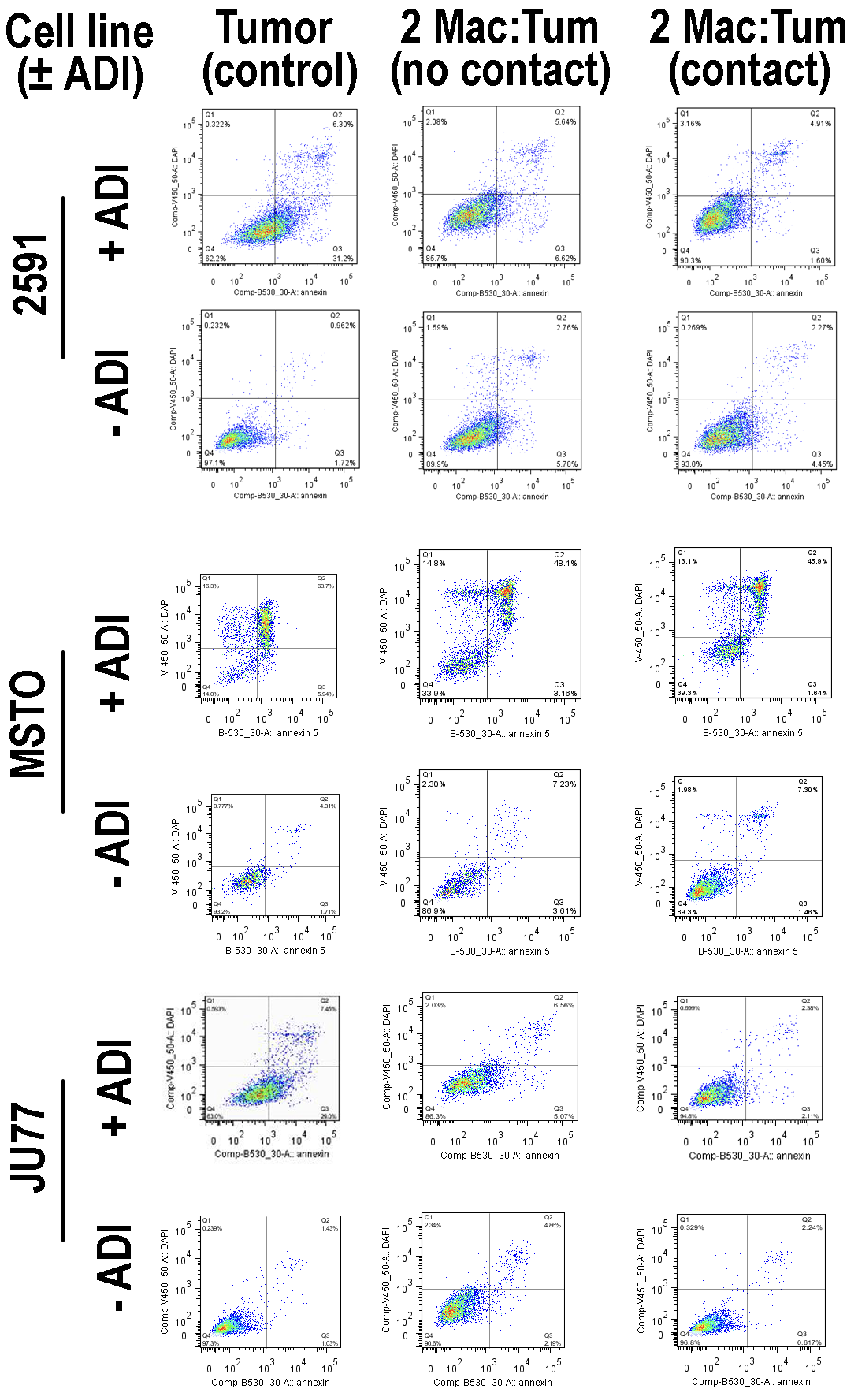

Supplement: Supplementary file 1 — Supplementary file1 (TIF 488 KB) [file 43440_2023_480_MOESM1_ESM.tif]
